# Association of daily and time-segmented physical activity and sedentary behaviour with mental health of school children and adolescents from rural Northeastern Ontario, Canada

**DOI:** 10.3389/fpsyg.2022.1025444

**Published:** 2022-10-25

**Authors:** Bruno G. G. da Costa, Brenda Bruner, Graydon H. Raymer, Sara M. Scharoun Benson, Jean-Philippe Chaput, Tara McGoey, Greg Rickwood, Jennifer Robertson-Wilson, Travis J. Saunders, Barbi Law

**Affiliations:** ^1^School of Physical and Health Education, Nipissing University, North Bay, ON, Canada; ^2^Department of Kinesiology, University of Windsor, Windsor, ON, Canada; ^3^Healthy Active Living and Obesity Research Group, Children’s Hospital of Eastern Ontario Research Institute, Ottawa, ON, Canada; ^4^Biotechnology Program & Functional Genomics and Clinical Consultation Program, Canadore College of Applied Arts and Technology, North Bay, ON, Canada; ^5^Department of Kinesiology and Physical Education, Wilfrid Laurier University, Waterloo, ON, Canada; ^6^Department of Applied Human Sciences, University of Prince Edward Island, Charlottetown, PE, Canada

**Keywords:** mental health, motor activity, public health, sedentary lifestyles, accelerometer

## Abstract

Physical activity (PA) and sedentary behaviour (SED) have been linked to the mental health of children and adolescents, yet the timing of behaviours may play a role in this relationship and clarifying this could inform interventions. We explored cross-sectional associations of PA and SED in varying time segments throughout the school day with the mental health of school-aged children and adolescents from rural Northeastern Ontario, Canada. A total of 161 students (56% female, M = 10.3 years old) wore accelerometers for 8 days (7 nights) and completed a self-report survey (parent reported for children younger than 11). Mental health was measured using the Strengths and Difficulties Questionnaire. Accelerometer-measured SED, light PA (LPA), and moderate and vigorous PA (MVPA) were estimated in the time-segments before school (06:00–08:44), school time (08:45–15:04), after school (15:05–16:59), and evenings (17:00–21:59). Associations were tested with multilevel linear regressions while adjusting for confounding factors. Students spent 72.6 min in MVPA, 209.0 min in LPA, and 621.0 min in SED per day. Daily SED was associated with less conduct problems (*β* = −0.27, *p* < 0.05). Evening LPA was inversely associated with hyperactivity (*β* = −1.45, *p* < 0.05), while SED was associated with hyperactivity and with peer problems before school (*β* = 1.70 and *β* = 1.01, respectively, *p* < 0.05), and during school (*β* = −0.83 and *β* = −0.57, respectively, *p* < 0.05). No associations were observed for MVPA, emotional symptoms, or prosocial behaviour. In conclusion, displacing SED with LPA in some specific periods of the day may benefit the mental health of students; taking this into account could strengthen interventions.

## Background

The mental health of children and adolescents has recently received increased attention in the scientific literature (Costello et al., 2005; Thapar et al., 2012), given a worrying increase in depressive symptoms and other indicators of mental ill-health in recent years. Unfortunately, an even worse scenario is projected by 2030, where depressive disorders are estimated to be the leading cause of burden of disease worldwide (World Health Organization, 2004). Among adolescents, depression is the major cause of disability across the globe and a strong risk factor for early mortality (Thapar et al., 2012; Petito et al., 2020; World Health Organization, 2021), and its onset during childhood and adolescence also predicts elevated risk of mental disorders in adulthood (Thapar et al., 2012; Mulraney et al., 2021). Mental health is multifactorial and although some of its biological risk factors are hard to change (Thapar et al., 2012), modifiable environmental (e.g., access to green space) and behavioural (e.g., physical activity, screen time) factors may be related with increased risk or protection (Schuch et al., 2018). Investigating modifiable risk factors associated with children and adolescents’ mental health is urgent, as interventions and policies to mitigate mental health problems must be informed by strong scientific evidence (Thapar et al., 2012).

There is an abundance of evidence showing that the maintenance of adequate levels of physical activity (PA) provides health benefits for pediatric populations (Janssen and Leblanc, 2010; Biddle and Asare, 2011; Rasberry et al., 2011; Poitras et al., 2016; Bidzan-Bluma and Lipowska, 2018; Whooten et al., 2019; Chaput et al., 2020), including the prevention of non-communicable diseases, improvement of mental health and academic performance, and ultimately better quality of life. Similarly, evidence has shown that sedentary behaviour (SED), or behaviours undertaken in a sitting or reclined position with low energy expenditure (Tremblay et al., 2017) have been linked to worse metabolic health, mental health, cognitive health, physical fitness, body composition, and holistic measures of health of children and adolescents (Tremblay et al., 2010, 2011; Chinapaw et al., 2011; Carson et al., 2016; Ekris et al., 2016; Biddle et al., 2017; Boberska et al., 2018), and should be avoided in excess. However, recent studies have shown that not all types of PA and SED are equal, and contextual factors may play an important role in their impact on health (Schmidt et al., 2017). Information about where each behaviour happens (e.g., in a park or at home), the types and contents of each behaviour (e.g., studying using a computer or using social media), and the timing of behaviours (e.g., during school hours or in the evening) are examples of contextual factors that may play a role in the relationship of PA and SED with mental health (Carson et al., 2016; Schmidt et al., 2017; da Costa et al., 2022a; Zou et al., 2022), and they have received little attention in scientific literature, possibly because of methodological challenges of measuring contextual factors, such as assessing time-stamped PA and SED. Although the role of different types of PA and SED in relation to mental health has been recently explored in the scientific literature, fewer studies have examined the timing of behaviour (i.e., when behaviour is happening) into account when analysing the relationship of PA and SED with mental health. A recent review with adults has found only three studies investigating the timing of PA and mental health, with two studies testing the impact of exercise in different periods of the day on mental health indicators of older adults, with one observing improvement in mood with exercise in the afternoon, and the other showing similar results between morning and evening exercises on mood; and one study comparing PA patterns between individuals with Alzheimer’s disease and healthy controls, observing significant differences in PA engagement in the morning (Janssen et al., 2022). Even less is known about how PA and SED in different time segments of the day relate to mental health of youth.

Previous studies found different associations between PA and SED participation and different domains of psychological difficulties measured by the Strengths and Difficulties Questionnaire (SDQ; Stone et al., 2015). Ahn et al. (2018) explored the relationship of accelerometer-measured PA and SED with SDQ-measured outcomes in school-aged children and found evidence that PA was inversely related to peer problems, and positively related to conduct problems and hyperactivity, whereas SED was positively related to peer problems and inversely related to hyperactivity scores (Ahn et al., 2018). In another study, self-reported PA was associated with increased prosocial behaviour and lower peer problems among boys and girls from London, United Kingdom (Brodersen et al., 2005), while SED was associated with increased conduct problems, hyperactivity, and emotional symptoms (Brodersen et al., 2005). Yet, these studies did not investigate the role of behaviours at different times of the day in relation to mental health.

Data derived from wrist-worn accelerometers provide a temporal record of PA that can be used to determine how SED and PA behaviours are distributed throughout the day (Sasaki et al., 2016), providing more contextual information such as how children spend their time before, during, and after attending school. However, it is unclear if the timing of behaviours is an important factor in the relationship between PA and SED with mental, social, or emotional health indicators. A study with Brazilian students (12.9 ± 5.3 years old) found no association between peer support for PA with PA of any intensity during school time, recess, or PE classes (da Costa et al., 2019a), but found a significant inverse relationship with SED during school time, suggesting that peer interactions such as encouragement and playing together are related to SED in this specific context (da Costa et al., 2019b). However, it is unclear if PA and SED during different periods of the day relate differently to conduct problems, peer problems, hyperactivity, emotional symptoms, and/or prosocial behaviour. Investigating how time-segmented PA and SED relate to different measures of mental health could inform interventions that specifically target PA and SED levels in one particular period of the day to maximize its impact on mental health indicators. Given the limited information on the relationship between accelerometer-derived data and different mental health outcomes among children and adolescents, the present cross-sectional study aimed to analyze the associations of PA and SED in different time-segments of the school day with mental health indicators in a sample of school children from Northeastern Ontario.

## Materials and methods

### Participants and procedure

Children and adolescents aged 5–14 were recruited from 3 rural elementary schools (K-8) in Northeastern Ontario (Canada) in May–June 2019 and January–February 2020, before it was interrupted by the COVID-19 pandemic. Following school board approval, an invitation to participate in the study was distributed by the school board to principals of rural schools within the board. The research team met with interested principals and school health promoters to explain the study. Upon agreement, study information was shared with teachers, and students were invited to participate. Each student received a package containing the study description, consent forms, and a survey link for their parents/guardians. Written informed consent was obtained from the participants’ legal guardians, and verbal assent was provided by each participant. The procedures of the study were approved by two local school boards and the Nipissing University Research Ethics Board.

### Measures

#### Physical activity and sedentary behaviour

Participants wore GENEActiv accelerometers (ActivInsights Ltd., Kimbolton, Cambridgeshire, United Kingdom) on their non-dominant wrist for 24-h across 8 days (7 nights). The GENEActiv is a small device (4.3 × 4.0 × 1.3 cm, 16 g) that resembles a wristwatch and measures acceleration between −8 g and 8 g across 3 axes (*x*, *y*, and *z*) with an 85.7 Hz sampling rate. After the data collection period, devices were retrieved by the research team, and data were downloaded using proprietary software (i.e., GENEActiv personal computer software version 3.2.). Raw accelerometer data were analyzed using the package GGIR (Migueles et al., 2019), according to the following steps: (1) autocalibration using local gravity as a reference (van Hees et al., 2014); (2) detection of sustained abnormally high values; (3) non-wear detection; and (4) calculation of the average magnitude of dynamic acceleration (Rowlands et al., 2016). The Euclidian norm minus one (ENMO) metric was the chosen data reduction method, using an epoch length of 5 s (van Hees et al., 2013).

ENMO thresholds validated for the pediatric population by Hildebrand and colleagues were used to calculate inactive (or stationary) behaviour (proxy of SED), and PA of light (LPA), and moderate-and-vigorous intensity (MVPA; Hildebrand et al., 2014, 2017). Quantification of SED and PA within predetermined time-segments of the day were calculated using a custom script written in MATLAB (MathWorks, Natick, MA, United States), using a daily class schedule provided by the schoolteachers. Time segments for waking behaviours were operationalized as Before school (06:00:00–08:44:55); School time (08:45:00–15:04:55); After School (15:05:00–16:59:55); and Evening (17:00:00–21:59:55). One of the three schools followed a balanced day schedule instead of traditional day schedule, with learning blocks organized in three periods of 100 min separated by two 40-min breaks for nutrition and play. The other two schools followed the traditional day schedule, with two shorter breaks in the morning and afternoon and a longer lunch break dividing the learning periods. For the balanced day school, the school time segment lasted 10 min longer, which was adjusted based on the threshold of the after-school period (i.e., the transition between the “School time” and “After School” segments was 15:15:00 instead of 15:05:00). Using the daily school schedule provided by the teachers, behaviours in physical and health education (PHE) classes, learning time, and recesses/breaks were also calculated. Data from holidays, school absences, and days when the school was closed due to inclement weather were not analyzed.

Accelerometer non-wear time was identified based on the standard deviation and value range of each axis being less than 13 and 50 mg, respectively (van Hees et al., 2013). Thus, the minimal accelerometer wear-time for inclusion in the data analyses was set at four valid days, with a valid day being defined as at least 16 h of accelerometer wear-time in the midnight-to-midnight period. For days with more than 16 h but less than 24 h of wear time, the difference was imputed using data from similar days. For the time-segmented analysis, a valid time segment was one in which at least 66% of its duration was classified as wear-time. All participants had to provide two or more valid segments to be included in the analyses of time-segmented data. The only exception was PHE classes, where the minimum number of valid PHE classes recorded was one, as some classes only had one PHE class per week. The median number of valid time segments per participant was 5 or above for all except PHE classes (median = 3).

#### Mental health

The SDQ (Goodman, 2001), a 25-item questionnaire, was used to measure five constructs of mental health: peer problems, hyperactivity, emotional problems, conduct problems, and prosocial behaviour. The SDQ was used in several previous studies with children and adolescents and its psychometric properties are considered strong in a review of validation studies, with a weighted (*n* = 53,691) mean internal consistency ranging from 0.53 to 0.81 across its multiple constructs for the parent-reported version and validated and confirmed factor structure (Goodman, 2001; Stone et al., 2010, 2015). Parents of children younger than 11 answered the SDQ for them, while children 11 and older self-reported. For each construct, the resulting scores range from 0–10 where higher values are unfavourable, except for prosocial behaviour, which is inverted (i.e., 10 means the worst score for conduct problems, but the best for prosocial behaviour).

#### Sociodemographic

Sex and date of birth were reported by the respondents (i.e., children or parents). Age was calculated. Both variables were used as covariates in statistical analyses.

### Analyses

Data were described using means and standard deviations. The internal consistency of latent variables measured with the SDQ was calculated using Cronbach’s Alpha and is presented in Supplementary Table 1.

To test the association of SED, LPA, and MVPA with mental health indicators, multilevel linear regression models were used. Since PA and SED within the same time segment are collinear (i.e., they must add up to the duration of a given class), models for SED, LPA, and MVPA were fit separately. For time-segmented data, a model was fit for each outcome and each behaviour but was mutually adjusted for the same behaviour in other time segments. For example, the association between MVPA and conduct problems included MVPA levels before school, at school, after school, and during the evening. This model structure was chosen since MVPA in different time segments are not necessarily collinear, but there may be some compensation, and thus were mutually adjusted (e.g., less MVPA in one segment due to higher MVPA in another). The data collection happened across different schools, grades, and different teachers and schedules, all of which are factors that can influence PA and SED levels. Thus, to address this variability, we used a multilevel structure with a random intercept for classes in which children are nested. Further, all models were adjusted for age and sex. Model residuals were inspected for heteroscedasticity, normality, and the effects of possible outliers. No transformations were made, or observations excluded.

The descriptive and inferential analyses were conducted in R, version 4.2.1 for Windows (R Foundation for Statistical Computing, Vienna, Austria). Statistical significance was set at *p* < 0.05 (two-tailed).

### Sensitivity analyses

The SDQ outcomes can also be expressed in summary measures of externalizing, internalizing, and total difficulties. Externalizing is the combination of the conduct and hyperactivity scales. Internalizing is the combination of emotional and peer problem constructs, and both range from 0 to 20 with 20 being the worst score possible. The total difficulties scale combines all constructs except for prosocial behaviour and varies between 0 and 40, also with the highest scores meaning more difficulties. As a sensitivity analyses, we also investigated of the associations of daily and time-segmented SED, LPA, and MVPA with internalizing, externalizing, and total difficulties, and the results are presented in Supplementary Tables 2, 3.

## Results

A total of 483 children and adolescents were invited to participate, and 279 (57.7%) were authorized by their guardians to participate. Of those, 161 (33.3% of the total invited) answered the survey questionnaire and provided valid accelerometer measures to be included in the analytic sample. The characteristics of the participants are described in Table 1. Participants were mostly female (approximately 56%), and were on average, 10.2 years old.

**Table 1:** Characteristics of the participants.

	Overall (*n* = 161)	Females (*n* = 90)	Males (*n* = 71)
	Mean (SD)	Mean (SD)	Mean (SD)
Age (years)	10.2 (2.30)	10.4 (2.14)	9.89 (2.48)
**SDQ variables**			
Emotional symptoms [0–10]	3.34 (2.43)	3.66 (2.43)	2.94 (2.39)
Conduct problems [0–10]	2.30 (1.65)	2.11 (1.46)	2.55 (1.84)
Hyperactivity-inattention [0–10]	4.89 (2.16)	4.88 (2.08)	4.90 (2.28)
Peer problems [0–10]	3.72 (1.45)	3.69 (1.47)	3.76 (1.44)
Prosocial behaviour [0–10]*	8.09 (1.80)	8.31 (1.72)	7.82 (1.87)
Total difficulties [0–40]	14.3 (5.74)	14.3 (5.56)	14.2 (6.00)

*Higher scores of prosocial behaviours are favourable.

SD, standard deviation; SDQ, strengths and difficulties questionnaire.

The time in minutes spent on MVPA, LPA, and SED on a habitual day and time segments of a school day are displayed in Table 2. Participants engage, on average, in 72.6 min of MVPA, 209.0 min of LPA, and 621.0 min of SED per day. In absolute terms, most of the daily MVPA was accumulated in the school time (43 min, on average), and participants spent approximately 20% of recesses and 23% of PHE in MVPA.

**Table 2:** Participant’s daily and time-segmented levels of SED, LPA, and MVPA (*n* = 161).

	Time segment	MVPA	LPA	SED
		Mean (SD)	Mean (SD)	Mean (SD)
Overall	Habitual day	72.6 (30.6)	209 (43.0)	621 (79.9)
	**Daily time-segments (minutes/segment)**			
	Before school (06:00–08:44)	9.15 (8.12)	24.9 (17.1)	135 (20.4)
	School time (08:45–15:04)	43.0 (17.1)	77.2 (20.0)	226 (40.9)
	After School (15:05–16:59)	16.6 (10.6)	31.5 (9.30)	73.2 (14.2)
	Evening (17:00–21:59)	30.9 (18.6)	55.8 (18.6)	161 (32.2)
	School time-segments (%/segment)			
	Recess time	0.197 (0.117)	0.263 (0.0630)	0.540 (0.155)
	PHE time	0.231 (0.145)	0.225 (0.0749)	0.545 (0.182)
Males	Habitual day	76.2 (30.1)	200 (42.9)	626 (76.0)
	**Daily time-segments (minutes/segment)**			
	Before school (06:00–08:44)	11.3 (9.28)	25.2 (15.9)	133 (19.5)
	School time (08:45–15:04)	45.6 (16.2)	76.0 (18.6)	223 (38.8)
	After School (15:05–16:59)	18.0 (10.3)	31.2 (9.56)	72.8 (14.0)
	Evening (17:00–21:59)	32.1 (19.3)	53.1 (19.1)	164 (34.2)
	School time-segments (%/segment)			
	Recess time	0.220 (0.118)	0.263 (0.0551)	0.517 (0.146)
	PHE time	0.246 (0.135)	0.219 (0.0677)	0.535 (0.165)
Females	Habitual day	69.7 (30.9)	216 (42.0)	618 (83.1)
	**Daily time-segments (minutes/segment)**			
	Before school (06:00–08:44)	7.45 (6.68)	24.6 (18.1)	136 (21.1)
	School time (08:45–15:04)	41.1 (17.7)	78.2 (21.0)	229 (42.5)
	After School (15:05–16:59)	15.6 (10.8)	31.8 (9.14)	73.6 (14.4)
	Evening (17:00–21:59)	30.0 (18.1)	58.0 (17.9)	159 (30.7)
	School time-segments (%/segment)			
	Recess time	0.179 (0.113)	0.262 (0.0688)	0.559 (0.161)
	PHE time	0.219 (0.153)	0.230 (0.0801)	0.552 (0.194)

MVPA, moderate-to-vigorous intensity physical activity; LPA, light-intensity physical activity; SED, sedentary behaviour; SD, standard deviation; PHE, physical and health education.

The association of daily PA and SED with emotional symptoms, conduct problems, hyperactivity-inattention, peer problems, and prosocial behaviours is shown in Table 3. A significant inverse association was observed only between SED with conduct problems (Coefficient: −0.27, 95% CI −0.49, −0.04).

**Table 3:** Association of daily levels of SED, LPA, and MVPA with mental health indicators (*n* = 161).

	Emotional symptoms [0–10]	Conduct problems [0–10]	Hyperactivity-inattention [0–10]	Peer problems [0–10]	Prosocial behaviour [0–10]†
	Coefficient (95% CI)*	Coefficient (95% CI)*	Coefficient (95% CI)*	Coefficient (95% CI)*	Coefficient (95% CI)*
MVPA (h/day)	0.06 (−0.73, 0.84)	0.24 (−0.28, 0.76)	−0.21 (−0.96, 0.54)	0.10 (−0.38, 0.58)	−0.01 (−0.57, 0.56)
LPA (h/day)	0.18 (−0.39, 0.74)	0.09 (−0.28, 0.46)	−0.36 (−0.88, 0.16)	0.12 (−0.22, 0.46)	0.01 (−0.40, 0.41)
SED (h/day)	0.06 (−0.27, 0.39)	**−0.27 (−0.49, −0.04)**	0.05 (−0.27, 0.36)	−0.03 (−0.23, 0.18)	0.14 (−0.10, 0.37)

*Unstandardized coefficients and 95% CI.

^†^The score for prosocial behaviour is inverted, and 10 is the best prosocial behaviour score possible.

MVPA, moderate-to-vigorous intensity physical activity; LPA, light-intensity physical activity; SED, sedentary behaviour.

Values in bold indicate statistically significant associations at *p* < 0.05.

Models were adjusted for sex and age.

The associations of time-segmented SED, LPA, and MVPA with emotional symptoms, conduct problems, hyperactivity-inattention, peer problems, and prosocial behaviours can be observed in Table 4. MVPA was not significantly associated with the outcomes. LPA during the evening was inversely related to hyperactivity-inattention (Coefficient: −1.45, 95% CI −2.81, −0.09). SED before school was related to increased hyperactivity-inattention (Coefficient: 1.70, 95% CI 0.16, 3.24) and increased peer problems (Coefficient: 1.01, 95% CI 0.02, 2.00), and was related to the same indicators during school time, but in the opposite direction (Coefficient: −0.83, 95% CI −1.56, −0.10; and Coefficient: −0.57, 95% CI −1.05, −0.09, respectively). In addition, an increased proportion of time spent on SED during PHE classes was also associated with fewer conduct problems (Coefficient: −1.55, 95% CI −3.02, −0.08) and more prosocial behaviour (Coefficient: 1.84, 95% CI 0.19, 3.50).

**Table 4:** Association of time-segmented SED, LPA, and MVPA with mental health indicators (*n* = 161).

	Emotional symptoms [0–10]	Conduct problems [0–10]	Hyperactivity-inattention [0–10]	Peer problems [0–10]	Prosocial behaviour [0–10]†
	Coefficient (95% CI)*	Coefficient (95% CI)*	Coefficient (95% CI)*	Coefficient (95% CI)*	Coefficient (95% CI)*
**Before school (06:00–08:44)**					
MVPA (h/segment)	−0.97 (−4.57, 2.64)	−1.07 (−3.37, 1.24)	−2.36 (−5.81, 1.09)	−0.82 (−2.91, 1.28)	0.09 (−1.15, 1.32)
LPA (h/segment)	−0.81 (−2.46, 0.85)	−0.38 (−1.41, 0.65)	−0.89 (−2.49, 0.71)	−0.32 (−1.24, 0.59)	−0.10 (−1.25, 1.05)
SED (h/segment)	0.58 (−1.10, 2.25)	0.52 (−0.65, 1.69)	**1.70 (0.16, 3.24)**	**1.01 (0.02, 2.00)**	−0.38 (−1.58, 0.82)
**School time (08:45–15:04)**					
MVPA (h/segment)	0.04 (−1.68, 1.76)	0.11 (−1.11, 1.32)	1.08 (−0.53, 2.69)	0.47 (−0.59, 1.53)	−1.71 (−3.55, 0.14)
LPA (h/segment)	−0.10 (−1.57, 1.36)	0.11 (−0.88, 1.11)	1.02 (−0.34, 2.38)	0.18 (−0.69, 1.06)	0.02 (−1.04, 1.08)
SED (h/segment)	−0.09 (−0.87, 0.70)	−0.19 (−0.76, 0.37)	**−0.83 (−1.56, −0.10)**	**−0.57 (−1.05, −0.09)**	0.17 (−0.39, 0.74)
**After School (15:05–16:59)**					
MVPA (h/segment)	−0.01 (−2.57, 2.56)	1.43 (−0.38, 3.25)	0.09 (−2.29, 2.47)	0.07 (−1.51, 1.65)	0.63 (−0.45, 1.71)
LPA (h/segment)	−0.32 (−3.19, 2.55)	1.08 (−0.98, 3.15)	2.00 (−0.66, 4.67)	0.61 (−1.17, 2.39)	−0.96 (−3.06, 1.13)
SED (h/segment)	0.34 (−1.71, 2.38)	−0.65 (−2.10, 0.80)	−0.79 (−2.65, 1.07)	−0.38 (−1.62, 0.86)	0.99 (−0.50, 2.47)
**Evening (17:00–21:59)**					
MVPA (h/segment)	0.45 (−1.06, 1.96)	−0.03 (−1.09, 1.03)	−0.77 (−2.19, 0.64)	−0.24 (−1.16, 0.69)	−0.63 (−0.45, 1.71)
LPA (h/segment)	0.88 (−0.57, 2.33)	0.00 (−1.04, 1.03)	**−1.45 (−2.81, −0.09)**	0.09 (−0.81, 0.99)	0.50 (−0.56, 1.56)
SED (h/segment)	−0.42 (−1.32, 0.49)	−0.13 (−0.76, 0.50)	0.82 (0.00, 1.64)	0.15 (−0.39, 0.69)	−0.41 (−1.07, 0.25)
**Recess time**					
MVPA (% of time)	- 2.06 (−5.31, 1.19)	−1.59 (−3.85, 0.68)	−1.49 (−4.69, 1.72)	0.36 (−1.64, 2.36)	0.06 (−2.35, 2.47)
LPA (% of time)	−3.42 (−9.44, 2.61)	−0.94 (−5.24, 3.36)	−1.27 (−4.69, 1.72)	1.14 (−2.59, 4.87)	−1.16 (−5.59, 3.26)
SED (% of time)	1.79 (−0.69, 4.28)	1.09 (−0.65, 2.83)	1.10 (−1.34, 3.55)	−0.41 (−1.94, 1.13)	0.17 (−1.68, 2.02)
**PHE time**					
MVPA (% of time)	0.20 (−2.61, 3.01)	1.61 (−0.24, 3.45)	0.47 (−2.13, 3.05)	0.25 (−1.44, 1.95)	−2.06 (−4.18, 0.07)
LPA (% of time)	2.50 (−2.58, 7.58)	2.81 (−0.64, 6.26)	2.64 (−1.96, 7.24)	1.90 (−1.19, 4.98)	−2.95 (−6.74, 0.84)
SED (% of time)	−0.60 (−2.79, 1.60)	**−1.55 (−3.02, −0.08)**	−0.77 (−2.78, 1.23)	−0.50 (−1.83, 0.82)	**1.84 (0.19, 3.50)**

*Unstandardized coefficients and 95% confidence intervals. Values where there is a significant association should be indicated by bold face.

^†^The score for prosocial behaviour is inverted, and 10 is the best prosocial behaviour score possible.

MVPA, moderate-to-vigorous intensity physical activity; LPA, light-intensity physical activity; SED, sedentary behaviour.

## Discussion

The present study aimed to analyze the associations of daily and time-segmented PA and SED with mental health indicators in a sample of school children and adolescents from rural Northeastern Ontario. In contrast to previous literature (Brodersen et al., 2005; Ahn et al., 2018; Bell et al., 2019), we only observed significant associations between daily SED and conduct problems. When time-segmented behaviours were investigated, SED was associated with hyperactivity, peer problems, prosocial behaviour, and conduct problems in specific segments, with some of these associations differing in direction depending on the time-segment analyzed. LPA was inversely associated with hyperactivity on the evening segment, and MVPA was not significantly associated with SDQ outcomes in any of the analyses.

Emotional symptoms were not related to SED, LPA, or MVPA for daily or time-segmented indicators in the present study, which contrasts with findings from previous studies (Brodersen et al., 2005; Bell et al., 2019). There are several potential explanations for this observed result, and a more recent study that used device-measured behaviours also found no association between PA and SED indicators with emotional symptoms among children (Ahn et al., 2018). Specifically for emotional symptoms, intrinsic values and experiences may be more relevant than the duration, intensity, and timing of PA and SED. Previous research shows that some specific types of PA may be more beneficial for quality of life; for example, sports may offer greater benefits than non-sports (da Costa et al., 2020a). Further, team sports may be more beneficial than individual sports for reducing the risk of anxiety and depression (Pluhar et al., 2019; Matias et al., 2022). Similarly, social media seems to be more detrimental to mental health compared to other types of screen-based SED (Boers et al., 2019; da Costa et al., 2022a). For other screen-based behaviours, such as watching videos or playing videogames, content related to violence can have a negative impact compared to other contents (Christakis and Zimmerman, 2007). Future research should consider both timing and the wider context of these behaviours to better understand children’s emotional wellbeing.

Only SED was significantly related to conduct problems, prosocial behaviour, and peer problems, while LPA during the evening was negatively associated with hyperactivity. An unanticipated finding was that the direction of the relationship of SED with hyperactivity and peer problems differed between the before-school and school time segments. While more SED before school was related to more peer problems and hyperactivity, SED also related to less problems when at school. The inverse relationship between SED at school and hyperactivity seems to be intuitive, as this relation was observed with a simultaneous positive relationship between PA and hyperactivity among children in a previous study (Ahn et al., 2018). Therefore, we expected to observe significant relationships of PA and SED with hyperactivity in different directions, but only the relationship with SED was observed. This is counterintuitive, as it was hypothesized that hyperactive children would be moving around more instead of being sedentary. Concerning both peer problems and hyperactivity, it is also unclear why the direction of their association with SED changed from before to during school. One possibility is that when children are more hyperactive, teachers may try to manage their behaviour by making the students stay in during recesses, and/or get sent to the office. In relation to peer problems, increased SED during school periods could be reflective of less physical interactions with peers or engagements that could escalate to conflicts, which sometimes happen during active play (da Costa et al., 2020b). Interactions and social dynamics during active play, class time and management, and commuting to school with peers may provide hints to why these relationships were segment-specific as well, and should be explored in future studies, as they have the potential to be changed to favour better mental health.

Children who spent more time on SED during PHE classes had less conduct problems and more prosocial behaviour. Considering that during physical activities such as teams sports and active play, interactions could include rough play and sometimes even conflicts (da Costa et al., 2020b), students who spend more time sedentary may be less exposed to such situations, resulting in less conduct problems and better prosocial behaviours. Furthermore, higher SED during PHE classes could also reflect the behaviours of students who are more compliant with teachers’ instructions and class management, compared to disruptive students, who may engage in lower prosocial behaviour and more conduct problems in this particular setting. However, considering that PHE is structured and guided by teachers, other factors such as social climate and norms may play a role in the interactions between peers and how it reflects on their behaviours and mental health (Morton et al., 2016). Some of these results might be also related to how some students respond to class management at the gym as opposed to the classroom, yet, our data does not allow for testing such hypotheses. Studies testing active interventions featuring martial arts have been shown to successfully reduce conduct problems, conflicts, and aggression in PHE classes (Carraro et al., 2014), suggesting that teacher training and changes in class may have the potential to improve the relations between students while also promoting PA. Combining direct observation and/or qualitative methods may also provide new insights into how to increase PA levels and promote mental health simultaneously in PHE classes.

Overall, high levels of MVPA were observed in the present study. Although children are normally depicted with low PA levels in large, survey-based studies (Hallal et al., 2012; Aubert et al., 2018; Guthold et al., 2020), research with accelerometry has also observed MVPA levels above 60 min per day among pediatric populations (Camiletti-Moirón et al., 2020; Ávila-García et al., 2021, Llorente-Cantarero et al., 2021; Padmapriya et al., 2021), which reflect the average recommended engagement in MVPA for children and adolescents according to the most recent World Health Organization guidelines and several international PA guidelines (Chaput et al., 2020; Parrish et al., 2020). We believe this to be a positive finding, considering the many health benefits that result from maintaining high levels of PA (Poitras et al., 2016; Chaput et al., 2020). However, we also recognize that high levels of PA in research with children and adolescents are often attributable to methodological decisions (Kim et al., 2017; Llorente-Cantarero et al., 2021; Leppänen et al., 2022). We have used standardized protocols and recommended acceleration cut-off points for the age-group of our sample (Migueles et al., 2017, 2019), and thereby, our results are comparable to several studies that used the same methods.

Although accelerometers excel at capturing intensity, duration, frequency, and timing of movement behaviours (Sasaki et al., 2016), which are robust predictors of several health outcomes such as cardiometabolic risk, body composition, and physical fitness (Poitras et al., 2016), they do not capture other contextual information that may be relevant for the mental health of children, such as types of physical activities/SED, with whom they are engaged, and aspects of the built, social, and political environments. Previous studies have shown that the relationship between PA and SED with mental health can change according to the measures of PA and SED (e.g., self-report versus accelerometers; Carson et al., 2016; Schmidt et al., 2017; da Costa et al., 2022a; Matias et al., 2022), as well as the correlates of accelerometer-measured PA and SED also differ according to the method of measurement (da Costa et al., 2021a,b). Self-reported instruments such as diaries are capable of investigating types of behaviours and other contextual information that may be more relevant for mental health and other subjective outcomes compared to intensity and duration (Piggin, 2020; da Costa et al., 2022a; Matias et al., 2022) that are stronger predictors of cardiometabolic and fitness outcomes (Poitras et al., 2016). The use of temporally resolved accelerometry data permits further analysis into how behaviours which occur at different times of the day are related to indicators of mental health. However, different activities that share similar intensities and postures may still affect mental health differently, such as playing videogames or using social media (da Costa et al., 2022b), or engaging in team or individual sports or other types of PA (Pluhar et al., 2019; da Costa et al., 2020a), or playing cooperatively or aggressively during recesses or during school time. Novel research methods such as combining wearable cameras and accelerometers may be needed to adequately assess intensity and duration of PA and SED while also collecting objective contextual information that can be used to further understand how they relate to mental health (Andriyani et al., 2022; Thomas et al., 2022).

The findings of the present study should be interpreted in light of potential limitations. First, the cross-sectional design of the study precludes causality of the associations observed. Although accelerometer-measured variables are time-stamped, the variability of mental health measures throughout the time-segments of day, which would allow for longitudinal analyses, are harder to assess. A second limitation is that the sample, which was selected by convenience, and whose size does not allow for more complex statistical techniques such as structural equation models. However, this sample represents an understudied population of school children and adolescents from rural northeastern Ontario, which differs from studies with samples from dense urban environments. Third, given that SED is commonly defined as any waking behaviour characterized by an energy expenditure less than or equal to 1.5 metabolic equivalents (METs) while in a sitting, reclining, or lying position, the accelerometry derived estimate of SED (i.e., stationary behaviour) in this study cannot be considered true SED, as it does not consider the posture component. However, stationary behaviour is highly correlated with SED, often being used as a proxy of it in studies with wrist-worn accelerometers. Notwithstanding the above, the strengths of the present study include the use of accelerometer-measured PA and SED, which are recommended among school-aged populations, and the use of a standardized comprehensive measure of mental health across different indicators.

## Conclusion

When overall behaviour levels were analyzed, only SED was associated inversely to conduct problems, yet LPA and SED in different time-segments of the school day were related to various indicators of mental health. In the evening LPA was favourably associated with hyperactivity, and SED was unfavourably associated with some indicators and favourably with others depending on the time of the day. MVPA was not associated with any mental health indicator, and emotional symptoms were not associated with any indicator of PA or SED. Taken together, displacing SED and PA in some periods of the day may be more beneficial for the mental health of students, yet future studies need to clarify other contextual information that may support creating welcoming and healthy engagement in different behaviours.

## Data availability statement

The datasets presented in this article are not readily available because authors are not authorized to share participants’ data. Requests to access the datasets should be directed to Dr. Barbi Law, barbil@nipissingu.ca.

## Author contributions

BGGC, BB, SMSB, and BL conceptualized the study. BB and BL secured funding for the study. BGGC, BB, BL, and GHR prepared the data and performed statistical analyses. BGGC, BB, and BL wrote the initial draft. All authors critically revised the manuscript. J-PC, TM, GR, JR-W, and TJS have equally contributed to this paper. All authors contributed to the article and approved the submitted version.

## Funding

This study is supported by the Canadian Institutes of Health Research (PJT-156209).

## Conflict of interest

The authors declare that the research was conducted in the absence of any commercial or financial relationships that could be construed as a potential conflict of interest.

## Publisher’s note

All claims expressed in this article are solely those of the authors and do not necessarily represent those of their affiliated organizations, or those of the publisher, the editors and the reviewers. Any product that may be evaluated in this article, or claim that may be made by its manufacturer, is not guaranteed or endorsed by the publisher.

## References

[ref1] Ahn J. V. Sera F. Cummins S. Flouri E. (2018). Associations between objectively measured physical activity and later mental health outcomes in children: findings from the UK millennium cohort study. J. Epidemiol. Community Health 72, 94–100. doi: 10.1136/jech-2017-209455, PMID: 29183954

[ref2] Andriyani F. D. Biddle S. J. H. Priambadha A. A. Thomas G. De Cocker K. (2022). Physical activity and sedentary behaviour of female adolescents in Indonesia: a multi-method study on duration, pattern and context. J. Exerc. Sci. Fit. 20, 128–139. doi: 10.1016/j.jesf.2022.02.002, PMID: 35308068 PMC8899402

[ref3] Aubert S. Barnes J. D. Abdeta C. Abi Nader P. Adeniyi A. F. Aguilar-Farias N.. (2018). Global matrix 3.0 physical activity report card grades for children and youth: results and analysis from 49 countries. J. Phys. Act. Health 15, S251–S273. doi: 10.1123/jpah.2018-0472, PMID: 30475137

[ref4] Ávila-García M. Esojo-Rivas M. Villa-González E. Tercedor P. Huertas-Delgado F. J. (2021). Relationship between sedentary time, physical activity, and health-related quality of life in Spanish children. Int. J. Environ. Res. Public Health 18:2702. doi: 10.3390/ijerph18052702, PMID: 33800169 PMC7967425

[ref6] Bell S. L. Audrey S. Gunnell D. Cooper A. Campbell R. (2019). The relationship between physical activity, mental wellbeing and symptoms of mental health disorder in adolescents: a cohort study. Int. J. Behav. Nutr. Phys. Act. 16:138. doi: 10.1186/s12966-019-0901-7, PMID: 31878935 PMC6933715

[ref7] Biddle S. J. H. Asare M. (2011). Physical activity and mental health in children and adolescents: a review of reviews. Br. J. Sports Med. 45, 886–895. doi: 10.1136/bjsports-2011-090185 21807669

[ref8] Biddle S. J. H. García Bengoechea E. Wiesner G. (2017). Sedentary behaviour and adiposity in youth: a systematic review of reviews and analysis of causality. Int. J. Behav. Nutr. Phys. Act. 14:43. doi: 10.1186/s12966-017-0497-8, PMID: 28351363 PMC5371200

[ref9] Bidzan-Bluma I. Lipowska M. (2018). Physical activity and cognitive functioning of children: a systematic review. Int. J. Environ. Res. Public Health 15:800. doi: 10.3390/ijerph15040800, PMID: 29671803 PMC5923842

[ref10] Boberska M. Szczuka Z. Kruk M. Knoll N. Keller J. Hohl D. H.. (2018). Sedentary behaviours and health-related quality of life. A systematic review and meta-analysis. Health Psychol. Rev. 12, 195–210. doi: 10.1080/17437199.2017.1396191 29092686

[ref11] Boers E. Afzali M. H. Newton N. Conrod P. (2019). Association of Screen Time and Depression in adolescence. JAMA Pediatr. 173, 853–859. doi: 10.1001/jamapediatrics.2019.1759, PMID: 31305878 PMC6632122

[ref12] Brodersen N. H. Steptoe A. Williamson S. Wardle J. (2005). Sociodemographic, developmental, environmental, and psychological correlates of physical activity and sedentary behavior at age 11 to 12. Ann. Behav. Med. 29, 2–11. doi: 10.1207/s15324796abm2901_2, PMID: 15677295

[ref13] Camiletti-Moirón D. Timperio A. Veitch J. Fernández-Santos J. D. R. Abbott G. Delgado-Alfonso Á.. (2020). Changes in and the mediating role of physical activity in relation to active school transport, fitness and adiposity among Spanish youth: the UP&DOWN longitudinal study. Int. J. Behav. Nutr. Phys. Act. 17:37. doi: 10.1186/s12966-020-00940-9, PMID: 32156288 PMC7063792

[ref14] Carraro A. Gobbi E. Moè A. (2014). Brief report: play fighting to curb self-reported aggression in young adolescents. J. Adolesc. 37:1303. doi: 10.1016/j.adolescence.2014.09.009, PMID: 25299437

[ref15] Carson V. Hunter S. Kuzik N. Gray C. E. Poitras V. J. Chaput J. P.. (2016). Systematic review of sedentary behaviour and health indicators in school-aged children and youth: an update. Appl. Physiol. Nutr. Metab. 41, S240–S265. doi: 10.1139/apnm-2015-0630, PMID: 27306432

[ref16] Chaput J. P. Willumsen J. Bull F. Chou R. Ekelund U. Firth J.. (2020). 2020 WHO guidelines on physical activity and sedentary behaviour for children and adolescents aged 5–17 years: summary of the evidence. Int. J. Behav. Nutr. Phys. Act. 17:141. doi: 10.1186/s12966-020-01037-z, PMID: 33239009 PMC7691077

[ref17] Chinapaw M. J. Proper K. I. Brug J. Mechelen W. Singh A. S. (2011). Relationship between young peoples’ sedentary behaviour and biomedical health indicators: a systematic review of prospective studies. Obes. Rev. 12, e621–e632. doi: 10.1111/j.1467-789X.2011.00865.x, PMID: 21438990

[ref18] Christakis D. A. Zimmerman F. J. (2007). Violent television viewing during preschool is associated with antisocial behavior during school age. Pediatrics 120, 993–999. doi: 10.1542/peds.2006-3244, PMID: 17974736

[ref19] Costello E. J. Egger H. Angold A. (2005). 10-year research update review: the epidemiology of child and adolescent psychiatric disorders: I. methods and public health burden. J. Am. Acad. Child Adolesc. Psychiatry 44, 972–986. doi: 10.1097/01.chi.0000172552.41596.6f, PMID: 16175102

[ref20] da Costa B. G. G. Barreto P. S. da Silveira P. M. da Silva J. A. Silva K. S. (2020a). The association between practicing sport and non-sport physical activities and health-related quality of life of Brazilian adolescents: a cross-sectional study. Sci. Sports 35, e109–e119. doi: 10.1016/j.scispo.2020.02.003

[ref21] da Costa B. G. G. Chaput J. P. Lopes M. V. V. Gaya A. R. Silva D. A. S. Silva K. S. (2021a). Association between sociodemographic, dietary, and substance use factors and accelerometer-measured 24-hour movement behaviours in Brazilian adolescents. Eur. J. Pediatr. 180, 3297–3305. doi: 10.1007/s00431-021-04112-0, PMID: 33993399

[ref22] da Costa B. G. G. Chaput J. P. Lopes M. V. V. Malheiros L. E. A. da Silva K. S.. (2021b). Associations between Sociodemographic, dietary, and substance use factors with self-reported 24-hour movement behaviors in a sample of Brazilian adolescents. Int. J. Environ. Res. Public Health 2021;18:2527. doi:10.3390/ijerph18052527, PMID: 33806301 PMC7967640

[ref23] da Costa B. G. G. Chaput J. P. Lopes M. V. V. Malheiros L. E. A. Silva K. S. (2022a). Movement behaviors and their association with depressive symptoms in Brazilian adolescents: a cross-sectional study. J. Sport Health Sci. 11, 252–259. doi: 10.1016/j.jshs.2020.08.003, PMID: 32791204 PMC9068734

[ref24] da Costa B. G. G. Chaput J. P. Silva K. S. (2022b). The two sides of sedentary behavior. J. Phys. Educ. 33. doi: 10.4025/jphyseduc.v33i1.3312

[ref26] da Costa B. G. G. Lopes M. V. V. Pizani J. Silva K. S. (2020b). Physical activity and sports participation associated with violence in adolescents: a systematic review. J. Phys. Educ. 31:e-3132.

[ref001] da Costa B. G. G. Silva K. S. Bandeira A. S. Martins C. R. Vieira J. A. J. Petroski E. L. (2019a). Pattern of sedentary behavior in different periods of school time of Brazilian adolescents. J. Sch. Health 89, 99–105. doi: 10.1111/josh.12716 30604452

[ref25] da Costa B. G. Silva K. S. da Silva J. A. Minatto G. de Lima L. R. Petroski E. L. (2019b). Sociodemographic, biological, and psychosocial correlates of light-and moderate-to-vigorous-intensity physical activity during school time, recesses, and physical education classes. J. Sport Health Sci. 8, 177–182. doi: 10.1016/j.jshs.2017.05.002, PMID: 30997264 PMC6450918

[ref27] Ekris E. Altenburg T. M. Singh A. S. Proper K. I. Heymans M. W. Chinapaw M. J. (2016). An evidence-update on the prospective relationship between childhood sedentary behaviour and biomedical health indicators: a systematic review and meta-analysis. Obes. Rev. 17, 833–849. doi: 10.1111/obr.12426, PMID: 27256486

[ref28] Goodman R. (2001). Psychometric properties of the strengths and difficulties questionnaire. J. Am. Acad. Child Adolesc. Psychiatry 40, 1337–1345. doi: 10.1097/00004583-200111000-00015 11699809

[ref30] Guthold R. Stevens G. A. Riley L. M. Bull F. C. (2020). Global trends in insufficient physical activity among adolescents: a pooled analysis of 298 population-based surveys with 1·6 million participants. Lancet Child Adolesc. Health 4, 23–35. doi: 10.1016/S2352-4642(19)30323-2, PMID: 31761562 PMC6919336

[ref31] Hallal P. C. Andersen L. B. Bull F. C. Guthold R. Haskell W. Ekelund U. (2012). Global physical activity levels: surveillance progress, pitfalls, and prospects. Lancet 380, 247–257. doi: 10.1016/S0140-6736(12)60646-1, PMID: 22818937

[ref32] Hildebrand M. Hansen B. H. van Hees V. T. Ekelund U. (2017). Evaluation of raw acceleration sedentary thresholds in children and adults. Scand. J. Med. Sci. Sports 27, 1814–1823. doi: 10.1111/sms.12795, PMID: 27878845

[ref33] Hildebrand M. Van Hees V. T. Hansen B. H. Ekelund U. (2014). Age group comparability of raw accelerometer output from wrist- and hip-worn monitors. Med. Sci. Sports Exerc. 46, 1816–1824. doi: 10.1249/MSS.0000000000000289, PMID: 24887173

[ref34] Janssen I. Campbell J. E. Zahran S. Saunders T. J. Tomasone J. R. Chaput J. P. (2022). Timing of physical activity within the 24-hour day and its influence on health: a systematic review. Health Promot. Chronic Dis. Prev. Can. 42, 129–138. doi: 10.24095/hpcdp.42.4.02, PMID: 35481335 PMC9116725

[ref35] Janssen I. Leblanc A. G. (2010). Systematic review of the health benefits of physical activity and fitness in school-aged children and youth. Int. J. Behav. Nutr. Phy. Act. 7:40. doi: 10.1186/1479-5868-7-40, PMID: 20459784 PMC2885312

[ref36] Kim Y. Hibbing P. Saint-Maurice P. F. Ellingson L. D. Hennessy E. Wolff-Hughes D. L.. (2017). Surveillance of youth physical activity and sedentary behavior with wrist accelerometry. Am. J. Prev. Med. 52, 872–879. doi: 10.1016/j.amepre.2017.01.012, PMID: 28526364 PMC5497761

[ref37] Leppänen M. H. Migueles J. H. Abdollahi A. M. Engberg E. Ortega F. B. Roos E. (2022). Comparing estimates of physical activity in children across different cut-points and the associations with weight status. Scand. J. Med. Sci. Sports 32, 971–983. doi: 10.1111/sms.14147, PMID: 35253276 PMC9311199

[ref38] Llorente-Cantarero F. J. Jurado-Castro J. M. Leis R. Vázquez-Cobela R. González-Gil E. M. Aguilera C. M.. (2021). Evaluation of sedentary behavior and physical activity levels using different accelerometry protocols in children from the GENOBOX study. Sports Med. Open. 7:86. doi: 10.1186/s40798-021-00365-z, PMID: 34817699 PMC8613328

[ref39] Matias T. S. Lopes M. V. V. da Costa B. G. G. Silva K. S. Schuch F. B. (2022). Relationship between types of physical activity and depression among 88, 522 adults. J. Affect. Disord. 297, 415–420. doi: 10.1016/j.jad.2021.10.051, PMID: 34715174

[ref40] Migueles J. H. Cadenas-Sanchez C. Ekelund U. Delisle Nyström C. Mora-Gonzalez J. Löf M.. (2017). Accelerometer data collection and processing criteria to assess physical activity and other outcomes: a systematic review and practical considerations. Sports Med. 47, 1821–1845. doi: 10.1007/s40279-017-0716-0, PMID: 28303543 PMC6231536

[ref41] Migueles J. H. Rowlands A. V. Huber F. Sabia S. Van H. V. T. (2019). GGIR: a research community–driven open source R package for generating physical activity and sleep outcomes from multi-day raw accelerometer data. J. Meas. Phys. Behav. 2, 188–196. doi: 10.1123/jmpb.2018-0063

[ref42] Morton K. L. Atkin A. J. Corder K. Suhrcke M. van Sluijs E. M. F. (2016). The school environment and adolescent physical activity and sedentary behaviour: a mixed-studies systematic review. Obes. Rev. 17, 142–158. doi: 10.1111/obr.12352, PMID: 26680609 PMC4914929

[ref43] Mulraney M. Coghill D. Bishop C. Mehmed Y. Sciberras E. Sawyer M.. (2021). A systematic review of the persistence of childhood mental health problems into adulthood. Neurosci. Biobehav. Rev. 129, 182–205. doi: 10.1016/j.neubiorev.2021.07.030, PMID: 34363845

[ref44] Padmapriya N. Chen B. Goh C. M. J. L. Shek L. P. C. Chong Y. S. Tan K. H.. (2021). 24-hour movement behaviour profiles and their transition in children aged 5.5 and 8 years: findings from a prospective cohort study. Int. J. Behav. Nutr. Phys. Act. 18:145. doi: 10.1186/s12966-021-01210-y, PMID: 34742314 PMC8572484

[ref45] Parrish A. M. Tremblay M. S. Carson S. Veldman S. L. C. Cliff D. Vella S.. (2020). Comparing and assessing physical activity guidelines for children and adolescents: a systematic literature review and analysis. Int. J. Behav. Nutr. Phys. Act. 17:16. doi: 10.1186/s12966-020-0914-2, PMID: 32041635 PMC7011603

[ref46] Petito A. Pop T. L. Namazova-Baranova L. Mestrovic J. Nigri L. Vural M.. (2020). The burden of depression in adolescents and the importance of early recognition. J. Pediatr. 218, 265–267.e1. doi: 10.1016/j.jpeds.2019.12.003, PMID: 31932020

[ref47] Piggin J. (2020). What is physical activity? A holistic definition for teachers, researchers and policy makers. Front. Sports Act. Living 2:72. doi: 10.3389/fspor.2020.00072, PMID: 33345063 PMC7739796

[ref49] Pluhar E. McCracken C. Griffith K. L. Christino M. A. Sugimoto D. Meehan W. P. (2019). Team sport athletes may be less likely to suffer anxiety or depression than individual sport athletes. J. Sports Sci. Med. 18, 490–496. PMID: 31427871 PMC6683619

[ref50] Poitras V. J. Gray C. E. Borghese M. M. Carson V. Chaput J. P. Janssen I.. (2016). Systematic review of the relationships between objectively measured physical activity and health indicators in school-aged children and youth. Appl. Physiol. Nutr. Metab. 41, S197–S239. doi: 10.1139/apnm-2015-0663, PMID: 27306431

[ref51] Rasberry C. N. Lee S. M. Robin L. Laris B. A. Russell L. A. Coyle K. K.. (2011). The association between school-based physical activity, including physical education, and academic performance: a systematic review of the literature. Prev. Med. 52, S10–S20. doi: 10.1016/j.ypmed.2011.01.027, PMID: 21291905

[ref52] Rowlands A. V. Yates T. Davies M. Khunti K. Edwardson C. L. (2016). Raw accelerometer data analysis with GGIR R-package: does accelerometer brand matter? Med. Sci. Sports Exerc. 48, 1935–1941. doi: 10.1249/MSS.0000000000000978, PMID: 27183118

[ref53] Sasaki J. E. da Silva K. S. da Costa B. G. G. John D. (2016). “Measurement of physical activity using accelerometers” in Computer-assisted and web-based innovations in psychology, special education, and health (Amsterdam, Netherlands: Elsevier), 33–60. doi: 10.1016/B978-0-12-802075-3.00002-4

[ref54] Schmidt S. C. E. Tittlbach S. Bös K. Woll A. (2017). Different types of physical activity and fitness and health in adults: an 18-year longitudinal study. Bio. Med. Res. Int. 2017:e1785217, 1–10. doi: 10.1155/2017/1785217, PMID: 28466006 PMC5390631

[ref55] Schuch F. B. Vancampfort D. Firth J. Rosenbaum S. Ward P. B. Silva E. S.. (2018). Physical activity and incident depression: a meta-analysis of prospective cohort studies. AJP 175, 631–648. doi: 10.1176/appi.ajp.2018.17111194, PMID: 29690792

[ref56] Stone L. L. Janssens J. M. A. M. Vermulst A. A. Van Der Maten M. Engels R. C. M. E. Otten R. (2015). The strengths and difficulties questionnaire: psychometric properties of the parent and teacher version in children aged 4–7. BMC Psychol. 3:4. doi: 10.1186/s40359-015-0061-8, PMID: 25815194 PMC4364334

[ref57] Stone L. L. Otten R. Engels R. C. M. E. Vermulst A. A. Janssens J. M. A. M. (2010). Psychometric properties of the parent and teacher versions of the strengths and difficulties questionnaire for 4- to 12-year-olds: a review. Clin. Child Fam. Psychol. Rev. 13, 254–274. doi: 10.1007/s10567-010-0071-2, PMID: 20589428 PMC2919684

[ref58] Thapar A. Collishaw S. Pine D. S. Thapar A. K. (2012). Depression in adolescence. Lancet 379, 1056–1067. doi: 10.1016/S0140-6736(11)60871-4, PMID: 22305766 PMC3488279

[ref59] Thomas G. Bennie J. A. Cocker K. D. Andriyani F. D. Booker B. Biddle S. J. H. (2022). Using wearable cameras to categorize the type and context of screen-based behaviors among adolescents: observational study. JMIR Pediat. Parent. 5:e28208. doi: 10.2196/28208, PMID: 35311672 PMC8981006

[ref60] Tremblay M. S. Aubert S. Barnes J. D.. (2017). Sedentary behavior research network (SBRN) – terminology consensus project process and outcome. Int. J. Behav. Nutr. Phys. Act. 14:75. doi: 10.1186/s12966-017-0525-8, PMID: 28599680 PMC5466781

[ref61] Tremblay M. S. Colley R. C. Saunders T. J. Healy G. N. Owen N. (2010). Physiological and health implications of a sedentary lifestyle. Applied physiology, nutrition, and metabolism =. Physiologie appliquee, nutrition et metabolisme. 35, 725–740. doi: 10.1139/h10-079 21164543

[ref62] Tremblay M. S. LeBlanc A. G. Kho M. E.. (2011). Systematic review of sedentary behaviour and health indicators in school-aged children and youth. Int. J. Behav. Nutr. Phys. Act. 8, 98–98. doi: 10.1186/1479-5868-8-98, PMID: 21936895 PMC3186735

[ref63] van Hees V. T. Fang Z. Langford J. Assah F. Mohammad A. da Silva I. C. M.. (2014). Autocalibration of accelerometer data for free-living physical activity assessment using local gravity and temperature: an evaluation on four continents. J. Appl. Physiol. 117, 738–744. doi: 10.1152/japplphysiol.00421.2014, PMID: 25103964 PMC4187052

[ref29] van Hees V. T. Gorzelniak L. León E. C. D.. (2013). Separating movement and gravity components in an acceleration signal and implications for the assessment of human daily physical activity. PLoS One 8:e61691. doi:10.1371/journal.pone.0061691, PMID: 23626718 PMC3634007

[ref64] Whooten R. Kerem L. Stanley T. (2019). Physical activity in adolescents and children and relationship to metabolic health. Curr. Opin. Endocrinol. Diabetes Obes. 26, 25–31. doi: 10.1097/MED.0000000000000455, PMID: 30507695 PMC6522241

[ref65] World Health Organization. (2004). The global burden of disease. Available at: https://apps.who.int/iris/bitstream/handle/10665/43942/9789241563710_eng.pdf (Accessed June 20, 2022).

[ref66] World Health Organization. (2021). Adolescent mental health. Available at: https://www.who.int/news-room/fact-sheets/detail/adolescent-mental-health (Accessed June 20, 2022).

[ref67] Zou Z. Xiang J. Wang H. Wen Q. Luo X. (2022). Association of screen time-based sedentary behavior and the risk of depression in children and adolescents: dose-response meta-analysis. Arch. Clin. Psychiatry (São Paulo). 48, 235–244. doi: 10.15761/0101-60830000000314

