# Supplementary material for: Association of daily and time-segmented physical activity and sedentary behaviour with mental health of school children and adolescents from rural Northeastern Ontario, Canada
Source: Front Psychol. 2022 Oct 25;13:1025444. doi: 10.3389/fpsyg.2022.1025444 (PMC9644206; doi:10.3389/fpsyg.2022.1025444)
Supplement: Supplementary file 1 [file Data_Sheet_1.docx]

| **Supplementary Table 1.** Internal consistency of the SDQ constructs (n = 161). | |
| --- | --- |
| **Construct** | Cronbach's Alpha (95%CI) |
| Emotional Symptoms | 0.71 (0.64-0.78) |
| Conduct Problems | 0.68 (0.60-0.75) |
| Hyperactivity-Inattention | 0.80 (0.75-0.85) |
| Peer Problems | 0.44 (0.32-0.57) |
| Prosocial Behaviour | 0.66 (0.58-0.74) |
| Total Difficulties | 0.84 (0.8-0.87) |

| **Supplementary Table 2.** Association of SED, LPA, and MVPA with mental health indicators. | | | |
| --- | --- | --- | --- |
|  | Total difficulties [0-40] | Internalizing | Externalizing |
|  | Coefficient (95%CI)* | Coefficient (95%CI)* | Coefficient (95%CI)* |
| MVPA (min/day) | 0.41 (-1.54, 2.36) | 0.16 (-0.90, 1.22) | 0.09 (-1.05, 1.22) |
| LPA (min/day) | 0.22 (-1.17, 1.60) | 0.26 (-0.50, 1.02) | -0.29 (-1.07, 0.48) |
| SED (min/day) | -0.29 (-1.10, 0.53) | 0.02 (-0.43, 0.46) | -0.27 (-0.74, 0.21) |
| *: Unstandardized coefficients and 95% confidence intervals. Values in bold indicate statistically significant associations at p < 0.05. Models were adjusted for sex and age. | | | |

| **Supplementary table 3.** Association of time-segmented SED, LPA, and MVPA with mental health indicators. | | | |
| --- | --- | --- | --- |
|  | Total difficulties [0-40] | Internalizing [0-20] | Externalizing [0-20] |
|  | Coefficient (95%CI)* | Coefficient (95%CI)* | Coefficient (95%CI)* |
| **Before school (06:00-08:44)** |  |  |  |
| MVPA (minutes) | -5.36 (-14.33, 3.61) | -1.70 (-6.48, 3.07) | -3.51 (-8.80, 1.78) |
| LPA (minutes) | -2.53 (-6.75, 1.69) | -1.28 (-3.51, 0.95) | -1.18 (-3.64, 1.29) |
| SED (minutes) | 3.75 (-0.36, 7.87) | 1.53 (-0.69, 3.75) | 2.17 (-0.25, 4.59) |
| **School time (08:45-15:04)** |  |  |  |
| MVPA (minutes) | 1.92 (-2.27, 6.11) | 0.49 (-1.83, 2.80) | 1.31 (-1.17, 3.80) |
| LPA (minutes) | 1.63 (-1.97, 5.23) | 0.21 (-1.77, 2.19) | 1.38 (-0.72, 3.48) |
| SED (minutes) | -1.69 (-3.59, 0.22) | -0.68 (-1.74, 0.37) | -1.03 (-2.16, 0.11) |
| **After School (15:05-16:59)** |  |  |  |
| MVPA (minutes) | 1.42 (-4.83, 7.67) | -0.04 (-3.50, 3.42) | 1.65 (-2.03, 5.33) |
| LPA (minutes) | 3.35 (-3.67, 10.36) | 0.26 (-3.60, 4.14) | 3.18 (-0.95, 7.31) |
| SED (minutes) | -1.51 (-6.45, 3.44) | -0.08 (-2.67, 2.83) | -1.65 (-4.55, 1.25) |
| **Evening (17:00-21:59)** |  |  |  |
| MVPA (minutes) | -0.37 (-4.06, 3.31) | 0.27 (-1.76, 2.30) | -0.76 (-2.94, 1.43) |
| LPA (minutes) | -0.25 (-3.84, 3.34) | 1.07 (-0.89, 3.03) | -1.42 (-3.52, 0.69) |
| SED (minutes) | 0.30 (-1.90, 2.50) | -0.24 (-1.45, 0.97) | 0.66 (-0.63, 1.95) |
| *: Unstandardized coefficients and 95% confidence intervals. | | | |
